# The effects of *Nigella sativa* on sickness behavior induced by lipopolysaccharide in male Wistar rats

**Published:** 2016

**Authors:** Fatemeh Norouzi, Azam Abareshi, Akbar Anaeigoudari, Mohammad Naser Shafei, Zahra Gholamnezhad, Mohsen Saeedjalali, Reza Mohebbati, Mahmoud Hosseini

**Affiliations:** 1 *Neurocognitive Research Center, School of Medicine, Mashhad University of Medical Sciences, Mashhad, Iran*; 2 *Pharmacological Research Center of Medicinal Plants, School of Medicine, Mashhad University of Medical Sciences, Mashhad, Iran*; 3 *Department of Physiology, School of Medicine, Jiroft University of medical Sciences, Jiroft, Iran*; 4 *Neurogenic Inflammation Research Center, School of Medicine, Mashhad University of Medical Sciences, Mashhad, Iran*; 5 *Mashhad Technical Faculty, Technical and Vocational University, Mashhad, Iran*

**Keywords:** *Rat*, *Lipopolysaccharide*, *Nigella Sativa*, *Sickness behavior*

## Abstract

**Objective:**

Neuroimmune factors contribute on the pathogenesis of sickness behaviors. *Nigella sativa* (NS) has anti-inflammatory, anti-anxiety and anti-depressive effects. In the present study, the effect of NS hydro-alcoholic extract on sickness behavior induced by lipopolysaccharide (LPS) was investigated.

**Materials and Methods:**

The rats were divided into five groups (n=10 in each): (1) control (saline), (2) LPS (1 mg/kg, administered two hours before behavioral tests), (3-5) LPS-*Nigella* *sativa* 100 , 200 and 400 mg/kg (LPS-NS 100, LPS-NS 200 and LPS-NS 400, respectively). Open- field (OF), elevated plus maze (EPM) and forced swimming test (FST) were performed.

**Results:**

In OF, LPS reduced the peripheral crossing, peripheral distance, total crossing and total distance compared to control (p<0.01- p<0.001). The central crossing, central distance and central time in LPS-NS 100, LPS-NS200 and LPS-NS 400 groups were higher than LPS (p<0.01- p<0.001). In EPM, LPS decreased the open arm entries, open arm time and closed arm entries while increased the closed time compared to control (p<0.001). Pretreatment by NS extract reversed the effects of LPS (p<0.05- p<0.001). In FST, LPS increased the immobility time while, decreased the climbing and active times compared to control (p<0.05- p<0.001). In LPS-NS 100, LPS-NS 200 and LPS-NS 400 groups the immobility time was less while, the active and climbing times were more than those of LPS (p<0.05- p<0.001).

**Conclusion:**

The results of the present study showed that the hydro-alcoholic extract of NS reduced the LPS-induced sickness behaviors in rats. Further investigations are required for better understanding the responsible compound (s) and the underlying mechanism(s).

## Introduction

Sickness behavior is characterized by features such as malaise, hyperalgesia, pyrexia, disinterest in social interactions, lethargy, behavioral inhibition, reduced locomotor activity, lower exploration and grooming behaviors, reduction of reproductive performance, anhedonia, somnolence, sleepiness, anorexia, weight loss, failure to concentrate, depression and anxiety (Maes et al, 2012 ▶). The behavioral features of sickness behavior can become visible following interaction between the surface molecules of the microorganisms such as bacteria and the receptors of the innate immune system cells which is accompanied by systemic inflammation and release of pro-inflammatory cytokines such as interleukin-1(IL-1), tumor necrosis factor (TNFα) and interleukin-6 (IL-6) (Burton et al, 2011 ▶). Enhancement of pro-inflammatory cytokines leads to shift metabolic energy from the brain and some peripheral organs to neutralize the effects of the invading pathogens through suppressing the energy consuming processes including locomotor, neurocognitive and reproductive activities (Maes et al, 2011 ▶). The second most common chronic disease, depression happens in children, adolescents, adults and elderly which is accompanied by sickness states such as sadness, loneliness, irritability, absurdity, despair, confusion, shame and the physical symptoms like reduction of locomotor activity (Sharp and Lipsky, 2002). The immune system stimulating factors have been proposed to be related to depression pathogenesis (Dantzer et al, 2011 ▶; Maes et al, 2009 ▶). For example, enhanced levels of the inflammatory markers such as C-reactive protein (CRP), haptoglobin, IL-6 and TNF-α have been reported to be associated with depression (Dowlati et al., 2010 ▶; Howren et al, 2009 ▶). Beneficial effects of anti-inflammatory and immunostimulating agents as an important strategy in the depression treatment confirm the role of inflammation in induction of depression and sickness behaviors (Janssen et al, 2010 ▶; Raison et al, 2006 ▶).

On the other hand, there is a relationship between the appearance of sickness behaviors and the brain levels of many neurotransmitters. Inadequate amount of some neurotransmitters particularly serotonin (5-hydroxytriptamine; 5HT) in the brain results in emersion of neurological disorder such as depression (Dantzer et al, 2011 ▶). It has been indicated that administration of tryptophan as a precursor of 5HT enhances the concentration of 5HT in the brain and induces anti-depression effects which in return can lead to improve sickness behaviors (Coppen and Doogan, 1988 ▶). 

A large number of medicinal plants such as *Nigella Sativa *(NS), a member of Ranuculaceae family, have been reported to have useful therapeutic effects (Pourbakhsh et al., 2014 ▶). It has been reported that NS increases the level of 5HT in the brain, improves learning and memory in rats and possesses anxiolytic effects (Ahmad et al., 2013 ▶). It has been demonstrated that thymoquinone (TQ), the main constituents of NS, produces antianxiety-like effects in mice through regulating the level of gamma-aminobutyric acid (GABA) and nitric oxide (NO) in the brain (Gilhotra and Dhingra, 2011 ▶). In addition, antioxidant effects of NS have been well documented. For example, NS oil showed antioxidant effects during cerebral ischemia-reperfusion in the hippocampal of rats (Hosseinzadeh et al, 2007 ▶). In addition, the extract of NS seeds and TQ exhibit anti-inflammatory effects through reducing the level of pro-inflammatory mediators such as IL-1β, IL-6, TNF-α, interferon-γ (IFN-γ) and prostaglandins and increasing anti-inflammatory cytokines such as interleukin-10 (IL-10) (Umar et al, 2012 ▶). 

Lipopolysaccharide ( LPS ) is an active component in cell membrane of gram-negative bacteria, which induces a systemic inflammation through promoting immune system cells and increasing the secretion of inflammatory cytokines including IL-1, IL-6, and TNF-α (Swiergiel and Dunn, 2007 ▶). Systemic injection of LPS induces a spectrum of behavior responses such as sickness behaviors which are along with the reduction of locomotor activity, exploration and feeding (Swiergiel and Dunn, 2007 ▶; Szentirmai and Krueger 2007 ▶; Azizi-Malekabadi et al 2015a ▶; Pourganj et al, 2014 ▶).

LPS has also induced a depression-like behavior when injected intracerebroventricularly (Lawson et al., 2013 ▶). Regarding the fact that inflammation induces a sickness-like behavior and also the improving effect of NS on anti-inflammatory responses and depression-like behaviors, the present study aimed to investigate the effect of the plant hydro-alcoholic extract on LPS-induced sickness behaviors in rats.

## Materials and Methods

**Animals and drugs**

Fifty male Wistar rats, 12-week old (240±10 g) were purchased from animal house of Mashhad University of Medical Sciences, Mashhad, Iran. The animals were kept under standard conditions (temperature 22±2 °C and 12 h light/dark cycle). The rats were allowed to use food and water freely. Working with the animals was fulfilled in accordance with approved procedures by the Committee on Animal Research of Mashhad University of Medical Sciences.

The animals were divided into five groups (n=10 in each group): (1) Control, (2) LPS, (3) LPS-NS extract 100 mg/kg (LPS-NS 100) , (4) LPS-NS extract 200 mg/kg (LPS-NS 200) and (5) LPS-NS extract 400 mg/kg (LPS-NS 400).

The animals in the LPS, LPS-NS 100, LPS-NS 200 and LPS-NS400 groups were treated with a single injection of LPS (1mg/kg; i.p.) 120 min before behavioral tests. The groups 3-5 received 100, 200 and 400 mg/kg of the extract (i.p.) 30 min before LPS administration (Hosseini et al 2015 ▶). The animals of control group received 1 ml/kg of saline instead of LPS. LPS was purchased from sigma (SigmaChemical Co).

**Behavioral tests**

*Open-field*

In open field test, after familiarization sessions with the apparatus environment, the rats were placed in the center of open field (100×100cm). The apparatus was placed in a room with a dim light. Movements were quantified using a digital camera for 5 min and the following parameters were calculated: (1) the number of crossing in the central zone, (2) the number of crossing in the peripheral zone, (3) the traveled distance in central zone,(4) the traveled distance in peripheral zone, (5) the time spent in central zone,(6) the time spent in peripheral zone, (7) the total crossing number, (8) the total traveled distance (Azizi-Malekabadiet al, 2015a ▶, Hosseini et al, 2012 ▶; Azizi-Malekabadi et al , 2015b ▶).

*Elevated plus maze*

A standard rat elevated plus maze with four arms (two open and two close arms) with 50 cm length and 10 cm width was used. The arms of the maze were roughly 100 cm above the floor. Each session which lasted 5 min was started by placing the rats in the central area facing the closed arms of the apparatus. After each session the apparatus was cleaned. The movements of the rats were quantified using a camera connected to computer. The time spent and the number of entries into the open and closed arms was determined (Azizi-Malekabadiet al, 2015a ▶ ; Azizi-Malekabadi et al, 2015b ▶).

*Forced swimming test*

Force swimming test was done for all groups as previously described (Azizi-Malekabadiet ala , 2015 ▶; Hosseini et al, 2012 ▶; Azizi-Malekabadi et alb , 2015 ▶). In summary, each rat was compelled to swim in a cylindrical glass tank (60 cm in hight and diameter of 38 cm) which was filled with water (40 cm depth) at 24±1 °C. The total duration of immobility was calculated by a single observer for 5 min. The immobility was considered when the rats made no effort to escape, except necessary movements which enabled them to keep their head above the water. The active and climbing times were also recorded for 5 min (Azizi-Malekabadiet al, 2015a ▶; Hosseini et al, 2012 ▶; Azizi-Malekabadi et al, 2015b ▶).

**Statistical analysis**

The data were expressed as mean ± SEM. One way ANOVA were run followed by tukey post-hoc comparisons test. The criterion for the statistical significance was p<0.05.

## Results

**Open-field**

**Figure1:**
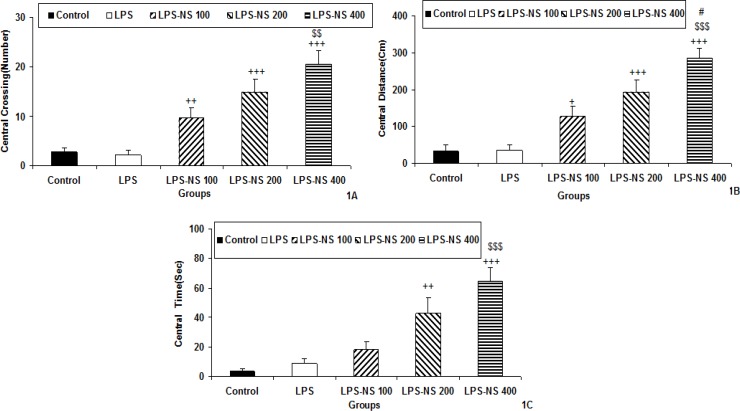
Comparison of central crossing number (A), the traveled distance in central zone (B) and the time spent in central zone (C) in the open field test between five groups. Data are expressed as mean ± SEM (n= 10 in each group).^ +^p<0.05,^++^p<0.01 and ^ +++^p<0.001 compared to LPS group, ^$$^p<0.01 and ^ $$$^p<0.001 compared to LPS-NS 100 group, ^ #^p<0.05 compared to LPS-NS 200 group

The results of open-field showed that there were no significant differences between LPS and control groups in the central crossing number (Figure 1A). The results also showed that the number of crossing in the central zone by the animals of LPS-NS 100 (p<0.01), LPS-NS 200 (p<0.01) and LPS-NS 400 (p<0.001) groups was significantly higher than that in the LPS group (Figure 1A). The animals of LPS-NS 400 group had also a higher crossing number in the central area compared to LPS-NS 100 group (p<0.01; Figure 1A).

There were no significant differences between LPS and control groups when the central-zone traveled distance was considered. However, the traveled distance in the central zone by the animals of LPS-NS 100 (p<0.05), LPS-NS 200 (p<0.001) and LPS-NS 400 (p<0.001) was higher than that of LPS group (Figure 1B). The animals of LPS-NS 400 group had also a higher traveled distance in the central area compared to LPS-NS 100 (p<0.001) and LPS-NS 200(p<0.05) groups (Figure 1B). 

The animals of LPS-NS 200 (p<0.01) and LPS-NS400 (p<0.001) group also spent more times in the central zone compared to LPS group. There were no significant differences between LPS and LPS-NS 100 groups in the time spent in the central zone (Figure 1C). The animals of LPS-NS 400 group (p<0.001) spent more time in the central area compared to LPS-NS100 group (Figure 1C). There were no significant differences between LPS and control groups in the time spent in the central zone (Figure 1C). 

**Figure 2:**
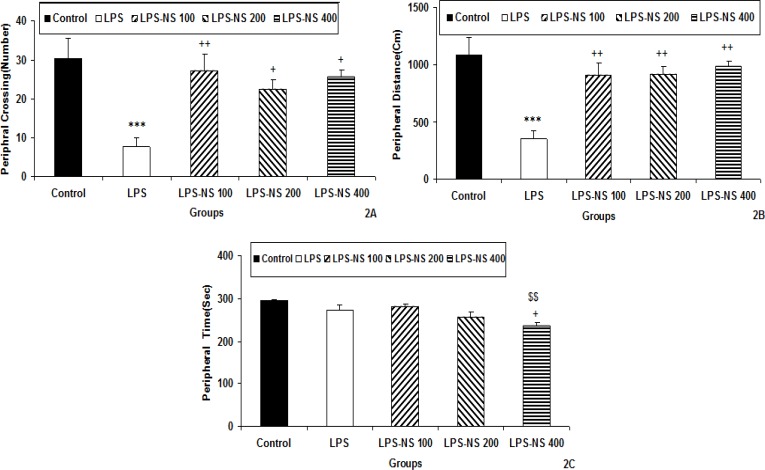
Comparison of peripheral crossing number (A), the traveled distance in peripheral zone (B) and the time spent in peripheral zone (C) in the open field test between five groups. Data are expressed as mean ± SEM (n= 10 in each group). *** p<0.001 compared to Control group.^ +^p<0.05 and^++^p<0.01 compared to LPS group,^ $$^p<0.01 compared to LPS-NS 100 group

The results of the open-field test also showed that the number of crossing in the peripheral zone in the LPS group was lower than that of the control group (p<0.001; Figure 2A). As shown in Figure 2A, the number of crossing in the peripheral zone in LPS-NS 100 (p<0.01), LPS-NS 200 (p<0.05) and LPS-NS 400 (p<0.05) groups was significantly higher than that in the LPS group. As the Figure 2B shows, the traveled distance in peripheral zone by the animals of LPS group was lower than that of control group (p<0.001). The animals of LPS-NS 100, LPS-NS 200 and LPS -NS 400 groups traveled longer distances in the peripheral zone compared to LPS group (p<0.01 for all; Figure 2B). There were no significant differences between the groups when the time spent in peripheral zone was compared between LPS and control groups and also when LPS-NS 100, LPS-NS 200 groups were compared with LPS group (Figure 2C). The peripheral time spent in the LPS-NS 400 group was less than that of LPS and group (p<0.05; Figure 2C). The animals of LPS-NS 400 group spent also less time in the peripheral zone compared to LPS-NS 100 group (p<0.01; Figure 2C).

**Figure 3:**
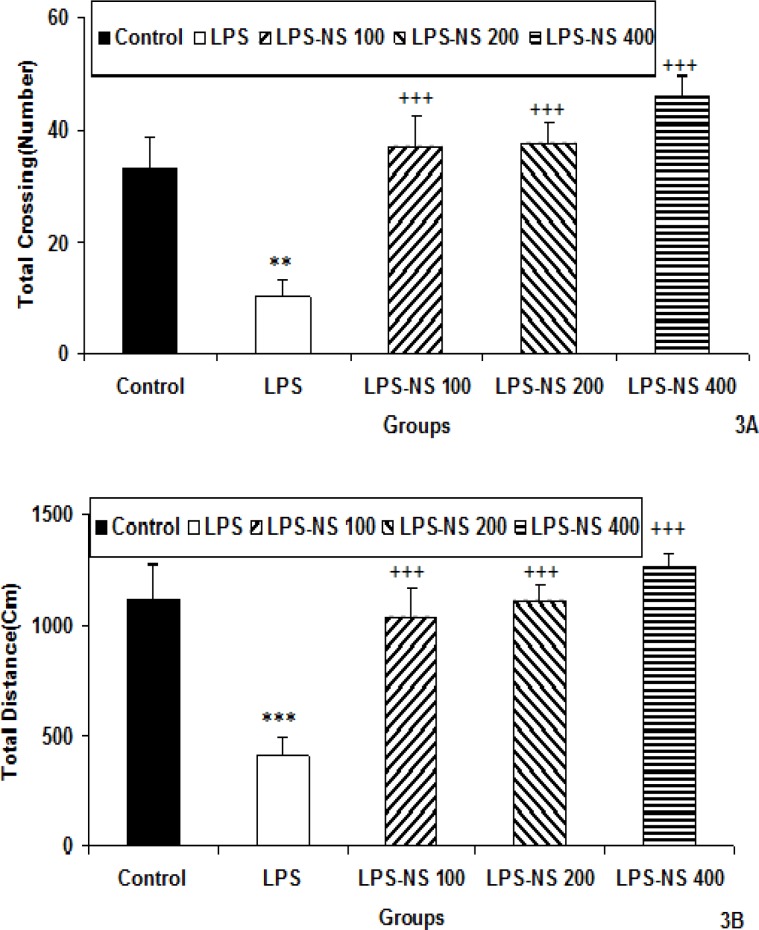
Comparison of total crossing number (A) and the total traveled distance (B) in the open-field test among five groups. Data are expressed as mean ± SEM (n=10 in each group). **p<0.01 and *** p<0.001 compared to Control group.^+++^p<0.001 compared to LPS group

The results of the open-field test also showed that the total number of crossing by the animals of LPS group was lower than that of the control group (p<0.01). As shown in Figure 3A, the total crossing number in LPS-NS 100, LPS-NS 200 and LPS-NS 400 groups was significantly higher than that in the LPS group (p<0.001 for all groups). As the Figure 3B shows, the total traveled distance by the animals of LPS group was less than that of control group (p<0.001). The total traveled distance by the animals of LPS-NS 100, LPS-NS 200 and LPS-NS 400 groups was more than that of LPS group (p<0.001 for all groups; Figure 3B).

**Elevated plus maze**

**Figure 4:**
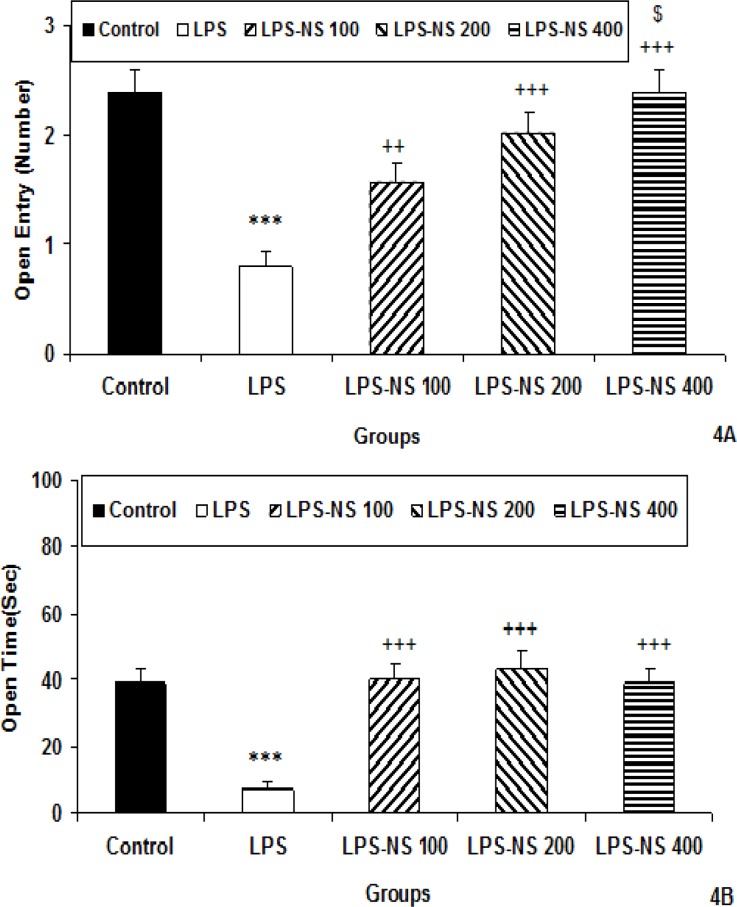
Comparison of the number of entries to the open arm (A) and the time spent in open arm (B) in the elevated plus maze test among five groups. Data are expressed as mean ± SEM (n= 10 in each group). *** p<0.001 compared to control group. ^++^p<0.01 and ^+++^p<0.001 compared to LPS group,^ $^p<0.5 compared to LPS-NS 100 group

In elevated plus maze, the number of entries to the open arm by the animals of LPS group was lower than that of control group (p<0.001; Figure 4A). In LPS-NS 100 (p<0.01), LPS-NS 200 (p<0.001) and LPS-NS 400 (p<0.001) groups, the number of entries to the open arm was higher than that of LPS group (p<0.01- p<0.001; Figure 4A).

The animals of LPS-NS 400 group had a higher number of entry to the open arm compared to the LPS-NS 100 group (p<0.05; Figure 4A).

The results also showed that the time spent in open arm by the animals of LPS group was less than that of control group (p<0.001; Figure 4B). The animals of LPS-NS 100, LPS-NS 200 and LPS-NS 400 groups spent more times in the open arm compared to LPS group (all p<0.001; Figure 4B). 

**Figure 5:**
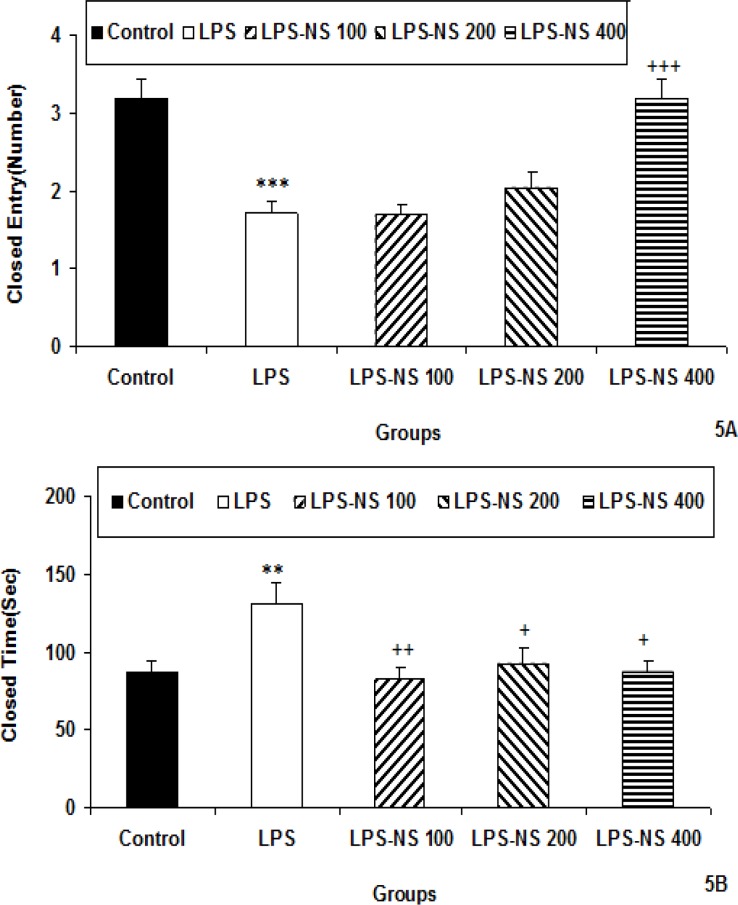
Comparison of the number of entries to the closed arm (A) and the time spent in closed arm (B) in the elevated plus maze test among five groups. Data are expressed as mean ± SEM (n= 10 in each group). **p<0.01 and ***p<0.001 compared to Control group +p<0.5, ++p<0.01 and +++p<0.001 compared to LPS group

The number of entries to the closed arm by the animals of LPS group was lower than that of control group (p<0.001; Figure 5A). There were no significant differences between LPS-NS 100, LPS-NS 200 and LPS groups in the number of entries to the closed arm while the number of entries to the closed arm by the animals of LPS-NS 400 was higher than LPS group (p<0.001; Figure 5A). The time spent in closed arm by the animals of LPS group was more than that of control group (p<0.01; Figure 5B). The animals of LPS-NS 100, LPS-NS 200 and LPS-NS 400 groups spent less times in the closed arm compared to LPS group (p<0.01, p<0.05 and p<0.05, respectively; Figure 5B). 

**Forced swimming test**

**Figure 6:**
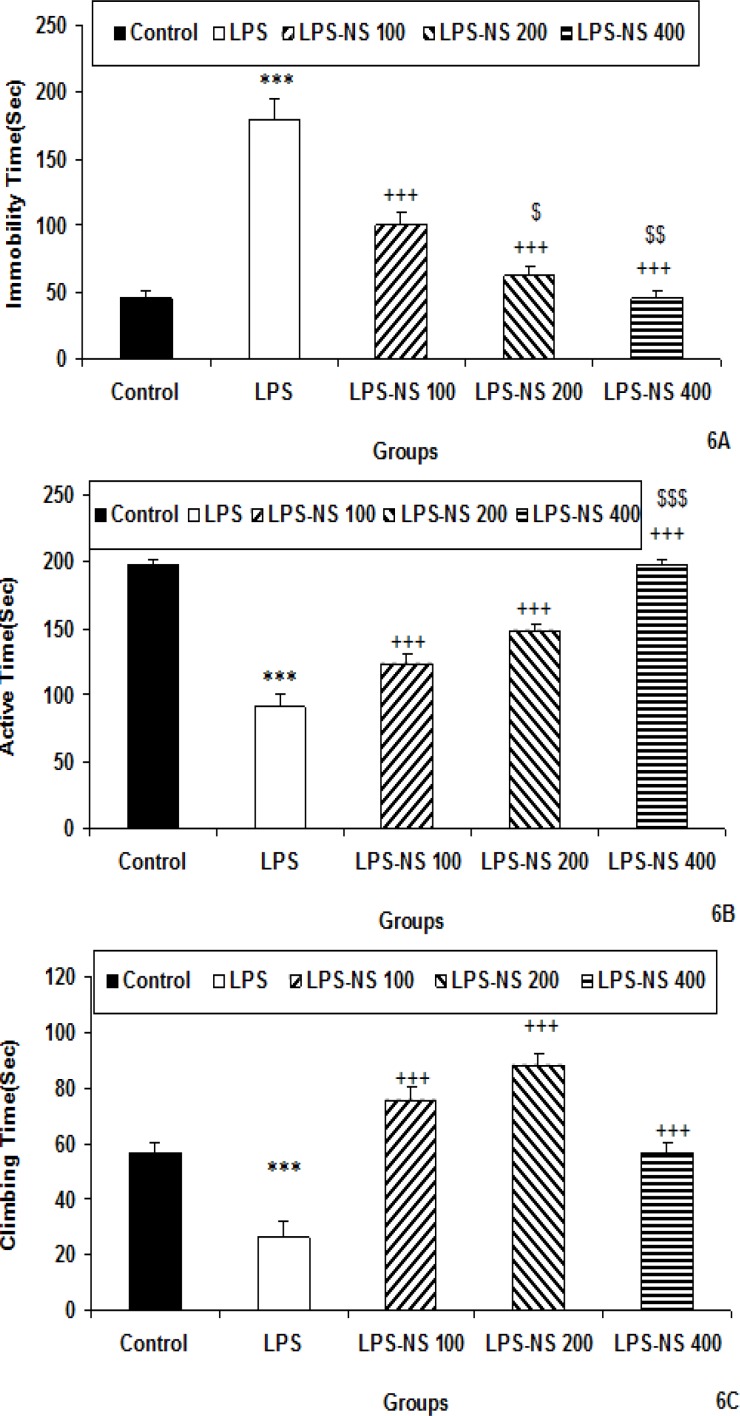
Comparison of immobility (A), active (B) and climbing time (C) in the forced swimming test among five groups. Data are expressed as mean ± SEM (n= 10 in each group). *** p<0.001 compared to control group. ^+++^p<0.001 compared to LPS group,^ $^p<0.05, ^$$^p<0.01 and ^$$$^p<0.001 compared to LPS-NS 100 group

The immobility times in the LPS group were more than that of the control group (p<0.001; Figure 6A). 

The immobility times in the LPS-NS 100, LPS-NS 200 and LPS-NS 400 groups were less than that of LPS group (p<0.001for all groups; Figure 6A). The results also showed that the animals of LPS-NS 200 and LPS-NS 400 groups had less immobility times compared to the LPS-NS 100 (p<0.05 and p<0.01, respectively; Figure 6A).

The results of force swimming test also showed that the active times in LPS groups was less than that of the control group (p<0.001; Figure 6B). The active times in the animals of LPS-NS 100, LPS-NS 200 and LPS-NS 400 groups were more than that of LPS group (p<0.001 for all groups; Figure 6B). The animals of LPS-NS 400 group had more active time compared to LPS-NS 100 (p<0.001; Figure 6B).

The climbing time in the LPS group was less than that of the control group (p<0.001; Figure 6C). The climbing times in the animals of LPS-NS 100, LPS-NS 200 and LPS-NS 400 groups were more than that of LPS group (p<0.001 for all groups; Figure 6C).

## Discussion

In the present study pretreatment with NS hydro-alcoholic extract improved sickness behaviors induced by LPS in rats. The sickness behaviors are resulted from activation of immune system following systemic microbial infections. Behavioral changes including decrease of food intake, social withdrawal, lethargy and disturbance in sleep-wake period overall called sickness behavior (Hosseini et al., 2012 ▶; Pourganji et al, 2014). LPS, as a biologically active component of the outer membrane of gram negative bacteria, is widely used in experimental animal models in order to induce systemic inflammation (Szentirmai and Krueger, 2014). LPS also stimulates the release of pro-inflammatory cytokines such as IL-1β, IL-6, and TNF-α in several areas of the brain including the hippocampus, hypothalamus and diencephalic structures when administered peripherally or centrally (Quan et al, 1994; Takao et al, 1993). LPS has also been frequently used in rodents to induce sickness behaviors which are presented by impairment of forced swimming, elevated plus maze and open field tasks (Azizi-Malekabadi et al, 2015a ▶; Burton et al, 2011 ▶). In the present study, LPS injection lead to an increase in immobility times in forced swimming time which was accompanied by decrease in active and climbing times. The force swimming test is a well-known tool for evaluation of depression-like behaviors in rodents (Azizi-Malekabadi et al, 2015b ▶; Neamati et al, 2014). It is also commonly used to measure the effectiveness of anti-depressants (Petit-Demouliere et al, 2005). Fluoxetine as a well known anti-depressant drug has been shown to be able to reduce immobility in forced swimming test (Contreras et al, 2001 ▶). A large number of evidence has confirmed the role of inflammation in depression. For example, treatment with interleukin-2 (IL-2) or IFN-γ in patients with cancer is accompanied by depressive symptoms (Miller, 2010 ▶; Piser, 2010). On the other hand, depressed patients exhibit all the cardinal features of inflammation (Miller, 2010 ▶). Various anti-inflammatory manipulations have also been suggested to have anti-depressant effects in experimental animals and humans (Leonard, 2001). IL-6 knockout mice showed a reduced depressive-like behavior in the forced swimming, tail suspension, learned helplessness, and sucrose preference tests (Chourbaji et al., 2006 ▶). Scientific studies have indicated that the IL-1 receptor knockout and administration of IL-1 receptor antagonist also suppress stress-induced depression-like behavior in the sucrose preference, social exploration tests, escape deficits, anhedonia and reduces social behavior (Maier and Watkins, 1995 ▶). In the present study, the corresponding depressive symptoms were observed in the LPS-treated rats. Previously, LPS has been indicated to induce sickness behaviors with behavioral symptoms including reduction in appetite and body weight, suppressed exploratory and social activities, fatigue and malaise, impairment of cognitive abilities, reduced libido and sexual behavior and anhedonia as well as depression-like behaviors (Kang et al., 2011 ▶). It has been previously reported that the effect of LPS may be seen even 28 days after injection (Layé et al., 2000 ▶). 

The results of present study showed that all three doses of extract prevented the depression and sickness behaviors induced by LPS. NS and its main active ingredient, TQ have been frequently shown to have immunostimulant, anti-tumor, anti-inflammatory and respiratory stimulant effects and have been shown to have some benefits in a number of diseases including allergy, colitis and bronchial asthma (Ali and Blunden, 2003 ▶; el Tahir et al, 1993 ▶; Hajhashemi et al, 2004 ▶; Houghton et al, 1995 ▶; Kalus et al., 2003 ▶; Mahgoub, 2003 ▶; Swamy and Tan, 2000; Worthen et al, 1998). The effect of NS on LPS- induced bronchial inflammation has also been reported (Entok et al., 2014 ▶). It was reported that TQ inhibits LPS-induced elevation of NF-κB, TNF-α, IL-1β, NO and PGE2, metalloproteinase-13 and cyclooxygenase-2 production (Vaillancourt et al., 2011; Wang et al, 2015). Regarding the provided evidence and the results of the present study, the beneficial effects of the plant extract on the sickness behaviors could be related to its anti-inflammatory properties. The results of open field test showed an increase in crossing number, as well as the time spent and the traveled distance in the central area of the apparatus also confirmed the results of anti-sickness effects of the extract. Moreover, the presence of the animals in the central area of open field which is considered as a sign of improvement of depression and sickness behaviors confirms the results of our study (Hosseini et al, 2012 ▶; Neamati et al, 2014).

LPS has been shown to increase norepinephrine and serotonin metabolism in the brain (Dunn, 2006 ▶) which play a crucial role in the activity regulation of effective brain area in emotion, including the limbic system (amygdala, hippocampus and nucleus accumbens), as well as the regulation of psychomotor functions and rewarding process including the basal ganglia (Dunn et al, 1999 ▶; Gao et al., 2002 ▶). Also, it has also been reported that NS increases the brain levels of 5-HT which might be considered as an explanation for the beneficial effects of the plant extract which were seen in the present study although more investigation should be undertaken in the future. Anxiety has been considered as a sign of sickness behaviors induced by LPS in rodents (Azizi-Malekabadi et al, 2015a ▶; Taksande et al, 2015). In the present study, LPS injection decreased the open entry as well as the time spent in the open arm of elevated plus maze test. It also increased the time spent in the closed arm of the maze. It has also been reported that LPS enhances the activity of the hypothalamic-pituitary-adrenal axis and increases sympathetic system activity (Dunn, 2000 ▶; Swiergiel and Dunn, 2007). These effects are similar to those happen during anxiety which might be considered as an explanation of anxiety-like behaviors after LPS injection as shown in the present study. Similar to our results, it has been previously reported that injection of LPS into substantia nigra is accompanied by anxiety-like behaviors in rats which was presented by decreasing in the percentage of the time spent in the open arms, the number of open-arm entries and the number of crossing in elevated plus maze (Hritcu and Gorgan, 2014 ▶). In the current study, NS extract showed anxiolytic effects presented by an increase in the entries and in the time spent in the open arm while, and a decrease in the time spent in the closed arm of elevated plus maze. The elevated plus maze is a well-known tool for evaluation of anxiety-like behaviors in rodents (Azizi-Malekabadi et al, 2015a ▶; Azizi-Malekabadi et al, 2015b ▶). Consistent with our results, it has been previously reported that repeated administration of NS decreases 5-HT turnover in the brains of rats to produce an anxiolytic effect (Perveen et al, 2009). This mechanism might have a role in the anxiolytic effects of the NS extract which was seen in the present study. However, it needs to be more investigated. 

NS has been reported to show a wide spectrum of activities and therapeutic effects (Ahmad et al, 2013 ▶). Protection against tissues oxidative damage and oxidative stress has been considered as an important mechanism (s) for the beneficial effects of the plant (Beheshti et al., 2014 ▶; M. Hosseini et al., 2014 ▶; Vafaee et al., 2015). Regarding these facts, protective effects against LPS-induced oxidative damage in brain tissues might be suggested as a possible explanation. In favor of this idea, we previously showed that LPS induces an oxidative damage in the hippocampal and the cortical tissues of the rats (Pourganji et al., 2014). In the present study, the component (s) responsible for the effects of the plant extract was not determined. Most of these effects have been frequently attributed to TQ as a major active chemical component in the plant. In addition to inhibition of cytokines release, administration of TQ has been shown to decrease lipid peroxidation, enhance the level of glutathione (GSH) and superoxide dismutase activity (Umar et al., 2012). In addition, TQ ameliorates LPS-induced hepatotoxicity in rats through decreasing MDA as the ultimate product of lipid peroxidation and normalizing GSH hepatic level when administered a long with LPS (Helal, 2010 ▶). The scientific finding confirmed that TQ recovers experimentally-induced arthritis in rats (Tekeoglu et al, 2007) and prevents the experimental autoimmune encephalomyelitis (Mohamed et al, 2005). Reports have also revealed that co-administration of TQ and LPS induces anti-inflammatory effects and reduces the serum level of cytokines such as TNFα and IL-1β (El Gazzar, 2007 ▶). Previously, it was indicated that TQ suppresses depression-like behavior induced by LPS (Hosseini et al, 2012 ▶). Considering this evidence, it might be suggested that TQ has an important role in the effects of NS which were seen in the present study however, it needs more investigations.

In the present study, NS improved open field impairments induced by LPS. In addition, high dose of NS extract decreased the time spent in the peripheral zone of the open field which may confirm the results of forced swimming and elevated plus maze tests reflecting the effects of the plant extract on depression and anxiety like behaviors and in general a sickness behavior induced by LPS. The open field has been frequently used to evaluate the anxiety and depression in the rodents. In these test, spending more time in peripheral is considered as a marker of anxiety and depression (Azizi-Malekabadi et al, 2015a ▶; Azizi-Malekabadi et al 2015b ▶; Neamati et al., 2014). It is also reported the drugs or natural products with anxiolytic and anti-depression effects increase the time spent by the animals in the central area while decrease the time in peripheral area (Azizi-Malekabadi et al, 2015a ▶; Azizi-Malekabadi et al 2015b ▶; Neamati et al., 2014). Furthermore, all doses of the extract increased the time and traveled distance as well as the crossing number in the central area which support the beneficial effects of the NS on sickness behavior induced by LPS. In the current study, injection of LPS (1 mg/ kg) reduced the crossing number and the traveled distance in peripheral zone of the open field which confirms sickness behavior inducing effects of LPS. It also reduced the total traveled distance and the total crossing number which is suggested to be resulted from its effect on the locomotor activity (Ma et al., 2011 ▶). The extract also increased the locomotor activities of the rats compared to LPS group which seems to be another evidence for beneficial effects of the plant extract.

The results of the present study confirmed the useful effects of NS on LPS-induced sickness behaviors caused by LPS in rats. The exact mechanism(s) and responsible compound(s) are needed to be investigated in future.
